# New Developments in the Use of Biologics and Other Modalities in the Management of Lateral Epicondylitis

**DOI:** 10.1155/2015/439309

**Published:** 2015-05-31

**Authors:** Cynthia A. Kahlenberg, Michael Knesek, Michael A. Terry

**Affiliations:** Department of Orthopaedic Surgery, Northwestern University Feinberg School of Medicine, 676 N. Saint Clair, Suite 1350, Chicago, IL 60611, USA

## Abstract

Lateral epicondylitis is a common source of elbow pain. Though it is often a self-limited condition, refractory lateral epicondylitis can lead to problems with activities of daily living and sometimes requires sick leave from work. Therefore prompt treatment is essential. Histopathologic studies have suggested that lateral epicondylitis is a tendinopathy, associated with apoptosis and autophagy, rather than a tendonitis associated with inflammation. Although corticosteroids have been used for short-term treatment, recent studies have suggested that they are not helpful and may even be harmful and delay healing in the treatment of lateral epicondylitis. Researchers have recently begun to investigate the use of biologics as potential treatment options for lateral epicondylitis. Autologous blood preparations including platelet rich plasma (PRP) and autologous whole blood injections (ABIs) have been proposed in order to deliver growth factors and other nutrients to the diseased tendon. Stem cell therapies have also been suggested as a method of improving tendon healing. This review discusses the current evidence for the use of PRP, ABI, and stem cell therapies for treatment of lateral epicondylitis. We also review the evidence for nonbiologic treatments including corticosteroids, prolotherapy, botulinum toxin A, and nitric oxide.

## 1. Introduction

Lateral epicondylitis, also known as “tennis elbow,” is a common cause of elbow pain in the adult population and affects 1-2% of the general public each year [1, 2]. In certain populations such as tennis players (9–40%) and physical laborers, the incidence is much higher [3]. Lateral epicondylitis is most commonly due to microtraumatic injury to the extensor carpi radialis brevis (ECRB) but may also involve other tendons within the forearm extensor muscles such as the extensor digitorum communis [4, 5].

Symptoms of lateral epicondylitis mainly consist of pain around the bony prominence of the lateral epicondyle of the elbow that radiates along the forearm within the area of the common extensor mass. The pain is typically exacerbated by contraction of forearm extensors with repetitive activities [6, 7]. Lateral epicondylitis is commonly a self-limiting condition that will resolve in approximately 90% of cases within one year without surgical intervention [8, 9]. However, Walker-Bone et al. [10] showed that 27% of patients with lateral epicondylitis reported severe difficulty with activities of daily living, and 5% of patients with lateral epicondylitis had taken sick leave from work, with an average duration of 29 sick days in the last 12 months due to their elbow symptoms. Thus, treatments for lateral epicondylitis are needed to help relieve patients' symptoms in a timely manner.

## 2. Pathophysiology of Lateral Epicondylitis

The pathology of lateral epicondylitis results from overuse of the extensor muscles leading to degenerative pathology of the involved tendons [9]. Although it is known as a “tendonitis,” histopathologic studies have shown that lateral epicondylitis is associated with few inflammatory type cells and is instead more associated with hypertrophy of fibroblasts, abundant disorganized collagen, hyperplasia of vascular elements, and eventually apoptosis and extracellular matrix breakdown [4, 11]. Furthermore, Alfredson et al. [12] found in a microdialysis investigation of ECRB tendons in patients with and without lateral epicondylitis that there was no increase in the local levels of the inflammatory mediator prostaglandin E2 in patients with lateral epicondylitis, which provides further evidence that the pathology of tennis elbow is not primarily an inflammatory response.

Nirschl defined four stages of damage caused by repetitive microtrauma involved in lateral epicondylitis of the elbow: (1) initial inflammatory reaction; (2) angiofibroblastic tendinosis, which refers to degenerative changes due to failure of a tendon to heal properly with presence of active fibroblasts, vascular hyperplasia, and production of disorganized collagen; (3) structural failure or rupture; and (4) structural failure plus fibrosis, soft matrix calcification, and hard osseous calcification [4, 13, 14]. Kraushaar and Nirschl [4] noted that when patients present with sports-related tendon injuries to the elbow such as lateral epicondylitis, they are most commonly at the stage of angiofibroblastic degeneration.

Further histopathologic studies have demonstrated that lateral epicondylitis and other tendinopathies are associated with apoptosis and autophagic tendon cell death, which contribute to cellular matrix breakdown [11, 15–17]. The lack of functional cells may impair normal collagen synthesis and the process of healing [11, 16]. It has been hypothesized that this impaired healing can lead to weaker tissue and susceptibility to further injury [16, 17]. In a model of a torn supraspinatus tendon, Tuoheti et al. [15] found a high rate of cellular apoptosis which they hypothesized may be due to the mechanical impingement leading to cell dysfunction and degeneration of the tendon. Chen et al. [11] found similar result of increased levels of apoptosis in the ECRB of patients with lateral epicondylitis. Chen and colleagues also found hypercellularity in their samples. They noted that their finding of hypercellularity in moderately damaged tendons in patients with lateral epicondylitis corresponded to the angiofibroblastic hyperplasia stage described by Nirschl and colleagues [4, 14]. They hypothesized that this hypercellularity represents an attempt at healing, but as the chronic damage to the tendon continues, the healing process becomes impaired by continued apoptosis and autophagy of remaining cells. Subsequently, the hypercellularity decreases, the synthesis of collagen and extracellular matrix components fails, and the tendon deterioration ensues [11].

When considering treatment modalities for patients with tendonopathy, it has been hypothesized that providing functional cells to the site of injury which are capable of synthesizing the extracellular matrix and repairing damage may help to overcome the apoptotic processes of tendinopathy and help restore tendon structure and function [18].

Several treatment options have been proposed to manage the pain and hasten the recovery for patients with lateral epicondylitis. While surgical options are available, most cases are amenable to less invasive treatment [9, 19]. Traditional conservative treatments have included rest, nonsteroidal anti-inflammatory drugs (NSAIDs), counterforce bracing, physical therapy, and corticosteroid injections [6]. Physical therapies employing eccentric rehabilitation protocols, which typically involve low intensity exercises at slow speeds with gradual intensification, have shown promising clinical results and are often the first line of treatment [3, 20]. Recent literature suggests that corticosteroid injections may actually have deleterious effects after their short-term pain relief [21, 22], and thus there has been a recent push to evaluate the possibility of biologics that may allow for healing of the chronic degeneration within the extensor tendons.

Therefore, researchers have begun to examine the role of biologics for management of lateral epicondylitis in an attempt to optimize the local environment for tendon healing and potential regeneration. This review will outline the current evidence for the use of biologics, specifically growth factors and mesenchymal stem cells, as well as the role of other commonly used modalities in the treatment of lateral epicondylitis.

## 3. Autologous Blood Preparations

Injections of autologous blood preparations, including platelet rich plasma (PRP) and autologous whole blood injections (ABIs), have gained popularity within the sports medicine literature because of their presumed safety and ease of use as a potential treatment for many musculoskeletal problems [23]. Tendons throughout the body, including those implicated in lateral epicondylitis such as the ECRB, heal more slowly than most other types of tissues partly due to the lower vascular supply [24, 25]. It is hypothesized that autologous blood preparations may help with healing because they initiate an inflammatory process while also delivering nutrients and high concentrations of growth factors that may promote tendon healing [7].

Autologous whole blood injections (ABIs), which involve withdrawing blood from an uninjured site and then reinjecting 2-3 milliliters into an area of injury or tendinopathy, have been widely studied for treatment of lateral epicondylitis (Table 1). Edwards and Calandruccio [26] hypothesized that ABI would initiate an inflammatory reaction around the tendon which would lead to cellular and humoral mediators that could induce a healing cascade and increase the rate of tendon repair. Other authors have hypothesized that the mechanism of ABI is essentially the same as PRP, as it allows for delivery of growth factors that can increase vascularity and new collagen formation for healing [27].

In their study of 28 patients with refractory lateral epicondylitis, Edwards and Calandruccio [26] found that, at an average follow-up of 9.5 months after ABI treatment, 79% had complete relief of pain, even during strenuous activity. Subsequently, Wolf et al. [28] investigated ABI in comparison to corticosteroid and saline injection in a randomized controlled multicenter trial and found that there were no significant differences in these three treatments at 6 months of follow-up. This study differed from other investigations in that it only included patients with lateral epicondylitis of less than 6-month duration and it excluded patients who had been treated with any type of injection therapy for lateral epicondylitis in the past 6 months.

Similar to ABIs, PRP is prepared using a sample of the patient's own blood. For PRP injections, however, this blood is then centrifuged to separate the liquid and solid components of the whole blood. In the first phase of centrifugation, which is conducted at 1,200–1,500 RPMs depending on the system utilized for preparation, plasma and platelets are separated from red and white blood cells. Subsequently, centrifugation is conducted at higher speeds (4,000–7,000 RPMs) to further concentrate the platelet rich components of blood [29]. In some cases, PRP preparations may also contain leukocytes including monocytes and polymorphonuclear neutrophils, which may help trigger a local inflammatory response and assist in the healing process for tendinopathy. However, the precise effect of various leukocytes on the healing process has not been fully defined [29].

PRP contains 3 to 10 times higher concentrations of platelets in comparison to autologous whole blood [30]. Platelets, which are tiny fragments of megakaryocytes that are formed in the bone marrow, have well-established roles in coagulation, inflammation, and immune modulation [31]. Degranulation of alpha granules contained inside platelets leads to release of platelet-derived growth factor (PDGF), vascular endothelial growth factor (VEGF), transforming growth factor- (TGF-) beta-1, insulin-like growth factor (IGF), epidermal growth factor (EGF), and fibroblast growth factor (FGF) [25, 29, 32–34].

PDGF has been shown to be influential in tendon healing by activating chemotaxis, proliferation of fibroblasts, collagen synthesis, and stimulation of other growth factors. TGF-beta-1 and IGF factor have been shown to increase collagen production, while certain types of VEGF (e.g., VEGF-111) stimulate angiogenesis in the otherwise relatively vascular environment of an injured tendon [25, 35]. FGF also plays a role in angiogenesis as well as cell migration, cell proliferation, and collagen synthesis at the tendon injury site [36]. In an in vitro study of the effect of PRP specifically on tenocytes, Zhang and Wang were able to demonstrate that PRP promotes differentiation of tendon stem cells (which are contained within normal human tendons) into tenocytes, which may aid in the healing process [37]. However, certain components of PRP including TGF-beta-1 have also been shown in animal models to inhibit bone healing and bone formation and may account for some of the variable outcomes of PRP treatments [29, 30, 38].

Upon administration of PRP, platelets begin to clot and subsequently secrete their growth factors within 10 minutes of clotting. The platelets also produce further quantities of these growth factors for several days after administration. Overall, in vitro studies of human tendons have demonstrated that PRP may stimulate cell proliferation, collagen production, and expression of matrix-degrading enzymes and endogenous growth factors, all of which help to accelerate organized remodeling and angiogenesis in the matrix of a damaged tendon and thereby may help to promote repair of tendon injury [25, 31]. After injection of PRP, an initial period of 7–10 days of resting the affected muscle/extremity is recommended, followed by eccentric rehabilitation therapy to maximize benefits of treatment and assist with healing [39].

Several major clinical studies have examined the role of PRP in the treatment of lateral epicondylitis (Table 1) [40–44]. One of the earliest investigations was a cohort study of 20 patients who had refractory lateral epicondylitis for a mean of 15 months and all of them were considering surgical intervention. Fifteen patients were given a single injection of PRP and five were given a single injection of bupivacaine. The authors found that, at 8 weeks after treatment, the PRP group had significantly lower pain compared to the bupivacaine group. Further follow-up of the PRP group at a mean of 25.6 months showed that these patients had a 93% reduction in pain compared to prior to treatment [40].

Similarly, a double-blinded randomized controlled trial of PRP injection versus corticosteroid injection for 100 patients with chronic lateral epicondylitis found that significantly more patients in the PRP group compared to the corticosteroid group had successful outcomes which were defined as greater than 25% reduction in visual analog score (VAS) of pain and Disabilities of the Arm, Shoulder, and Hand (DASH) score at 1 year after intervention [42]. The same group of authors reported the outcomes of this trial at two years after treatment and found that, at that time, patients treated with PRP continued to have reduced pain and better function compared to the corticosteroid group [43]. This randomized controlled trial was criticized in the literature for the use of corticosteroid as the control, as it has been hypothesized that since lateral epicondylitis is not an inflammatory process, corticosteroid would have no benefit and may even be harmful, in the treatment of this condition [41, 44].

Krogh et al. [44] conducted a double-blinded randomized controlled trial of PRP versus glucocorticoid injection versus saline injection for 60 patients with chronic lateral epicondylitis and showed no significant difference between PRP, glucocorticoid, and saline for pain reduction at the study endpoint of 3 months. However, there was no data reported after 3 months of follow-up, which could possibly account for the discrepancy in results in comparison to prior trials. More recently, Mishra et al. [41] reported the results of a multicenter double-blinded randomized controlled trial of injection of PRP versus bupivacaine in 230 patients with chronic lateral epicondylitis and found no significant differences at 12 weeks but did find a significant reduction in pain in the PRP treatment group at 24 weeks after injection.

Treatment with PRP versus ABI was directly compared in a randomized controlled trial by Thanasas et al. [45]. They found a greater improvement in VAS scores in patients treated with PRP compared to ABI at 6 weeks of follow-up but found no significant differences between these treatments at 3 months or 6 months after injection. Creaney et al. [27] conducted a similar randomized controlled trial of PRP versus ABI for treatment of refractory lateral epicondylitis. The authors demonstrated a 66% success rate in the PRP group versus 72% success rate in the ABI group, which was not statistically significantly different. They argued that both of these autologous blood preparations (PRP and ABI) might be effective as second line therapy for treatment of refractory lateral epicondylitis. In addition, Raeissadat et al. conducted a one-year randomized controlled trial of ABI versus PRP injection and found significant improvements in both groups but no statistically significant differences between the groups [46]. Given similar results using PRP versus ABI, the authors hypothesized that there may be a threshold effect with the delivery of growth factors and that delivery of increased concentrations (as is the case with PRP) may not be more beneficial than the delivery of growth factors through ABI because physiological mechanisms of healing may be saturated and already be driven to work at maximum capacity with the growth factors delivered through whole blood injection [27].

No significant adverse events were noted in any of these studies [40–44], though the study by Krogh et al. [44] did find that PRP injection caused more postinjection pain compared to saline or glucocorticoid. Furthermore, Kaux et al. reported one case of an exuberant inflammatory reaction to PRP injection in a type 1 diabetic patient treated with PRP for patellar tendinopathy in which the patient experienced pain, erythema, heat, and swelling at the injection site and two weeks later he was found to have thickening of the tendon on ultrasound [47]. Overall, however, reported adverse events related to PRP are extremely rare in the literature.

A major limitation in the evaluation of the efficacy of PRP in treatment of lateral epicondylitis and other musculoskeletal diseases is the heterogeneity in the way with which PRP is prepared and administrated [29, 31, 33]. This heterogeneity of PRP preparations may account for the variable outcomes noted in the prior studies evaluating efficacy of PRP for lateral epicondylitis. Several groups are now working to find a method of standardizing the preparation of PRP, which will be greatly beneficial for future research and application of this treatment.

Overall, autologous blood preparations including PRP and ABI have shown variable results in randomized controlled trials but have shown some promise in the treatment of refractory lateral epicondylitis. Future studies with long-term follow-up, larger patient groups, and a standardized method for preparation of the injection are needed to better define the efficacy of these treatments for lateral epicondylitis.

## 4. Stem Cells and Related Therapies

A more recent development in the field of biologics for the treatment of lateral epicondylitis is the use of mesenchymal stem cells and related therapies. As previously discussed, studies of tendinopathy have shown increased rates of apoptosis, leading to a lack of cellular components in the area of an injured tendon which impairs the healing response [11, 16]. The depletion of tenocytes via apoptosis has specifically been shown to impair collagen synthesis and tendon healing [18, 48].

Chen et al. [48, 49] have shown that it is feasible to conduct in vitro multiplication of primary tenocytes; furthermore, injection of these autologous tenocytes improves tendon remodeling, histological outcomes, collagen content, and tensile strength in a degenerative Achilles tendon tear in a rabbit model. Given that elevated rates of apoptosis and autophagic cell death have been observed in the ECRB tendons of patients with chronic lateral epicondylitis [11], recent studies have attempted to look at ways to directly deliver functional cells that are capable of synthesizing extracellular matrix and repairing damaged tissue to the area of tendon injury. The three types of tissue that have been utilized for tendon repair include multipotent stem cells, skin fibroblasts, and tenocytes. Of note, a study by Harris et al. [50] has raised concern about the use of multipotent stem cells of mesenchymal origin due to their potential to differentiate into osteoblastic cells and cause ectopic bone formation instead of tendon healing.

In a pilot study of 12 patients with refractory lateral epicondylitis, Connell et al. [51] demonstrated that collagen-producing tenocyte-like cells derived from autologous skin fibroblasts that were injected into the elbow with a platelet rich plasma matrix for patients with refractory lateral epicondylitis provided both clinical and structural improvements (Table 1). The median Patient-Rated Tennis Elbow Evaluation (PRTEE) score showed significant improvement at 6 weeks, 3 months, and 6 months after injection. On ultrasound, the common extensor tendon origins of patients in the study appeared to have undergone restoration after the treatment, based on a decrease in tendon size, restoration of the fibrillar pattern, decreased neovascularization, and near-total resolution of intrasubstance tears.

Recently, Wang et al. [18] published a pilot study of the use of autologous tenocytes derived from patellar tendon cells which were expanded in vitro and injected under ultrasound guidance near the lateral epicondyle for treatment of severe refractory lateral epicondylitis in 16 patients. Tendon-derived cells were chosen for this investigation because of their potential for collagen synthesis, rapid proliferation, and self-renewability. At each reported follow-up point (1 month, 3 months, 6 months, and 12 months), patients had a significant reduction in pain on the VAS scale as well as improved Quick-DASH scores and grip strength scores. The study also evaluated each patient on MRI and found that there was significant improvement in structural repair at the common extensor tendon origin [18].

In an attempt to combine the benefits of PRP and stem cell technologies, Singh et al. [52] conducted a pilot study of the efficacy of treatment of lateral epicondylitis using a bone marrow aspirate containing both PRP and mesenchymal stem cells. A similar injection was previously studied in patients with patellar tendinopathy and showed statistically significant clinical improvement in a small cohort at 5 years of follow-up [53]. For lateral epicondylitis, Singh et al. found a significant improvement in PRTEE scores at two weeks, six weeks, and twelve weeks after treatment. Of note, the patients included in this series had a mean duration of symptoms of only 7.33 weeks and had no prior attempted treatments [52].

Although these emerging stem cell technologies show promise for the treatment of refractory lateral epicondylitis, only pilot studies are available at this point. Larger-scale randomized controlled trials are needed to compare the efficacy of stem cells to other standard treatments for management of lateral epicondylitis and also to establish the long-term safety of these interventions.

## 5. Botulinum Toxin A

Botulinum toxin A is another modality that has been suggested for the treatment of lateral epicondylitis. It works via blocking the acetylcholine receptor in the presynaptic junction causing a temporary palsy within the skeletal muscle. Morré and colleagues [54] first described its utilization for lateral epicondylitis in 1997. Authors suggest that botulinum toxin injections help in the treatment of lateral epicondylitis by causing a reversible paralysis to the extensors, particularly ECRB and extensor carpi radialis longus (ECRL), which prevents further microtrauma to the origin and allows the pathologic tissue to heal [6, 55, 56]. Several studies have demonstrated promising results in the use of botulinum toxin A for treatment for lateral epicondylitis. Placzek and colleagues [56] performed a multicenter double-blinded randomized controlled trial whereby 130 patients were treated with either botulinum toxin or placebo and those treated with botulinum had significantly improved VAS and clinical pain scores at 6, 12, and 18 weeks. However, the induced paralysis may have some significant clinical consequences, with some studies demonstrating weakness within the wrist extensors and with grip strength after treatment with botulinum. Lin and colleagues [57] performed a double-blinded randomized controlled trial between botulinum and corticosteroid injection and found significantly decreased pain scores in the patient group treated with corticosteroid compared to botulinum. However grip strength was consistently lower within the botulinum group. Overall, the current evidence available regarding botulinum A toxin in the treatment of lateral epicondylitis is inconsistent and further studies are needed to determine if this is an effective treatment modality.

## 6. Nonbiologic Injection Therapy

### 6.1. Corticosteroid

For many years, injection of corticosteroid has been a common treatment for patients with refractory lateral epicondylitis. Corticosteroids work by decreasing the inflammatory cascade and suppressing the local immune response to pain, which was thought to be important for the treatment of lateral epicondylitis. As the scientific community has grown in the understanding of epicondylitis being more of tendinosis without significant amounts of inflammatory cells, studies have found increased levels of substance P (neurokinin-1) receptors in these patients [58]. Studies suggest that corticosteroids may reduce substance P levels elsewhere in the body, and thus this is a proposed mechanism by which they may provide pain relief in epicondylitis [59]. Dexamethasone, betamethasone, and triamcinolone are all used and commonly are mixed with a local anesthetic such as lidocaine or bupivacaine. Earlier studies suggested greater benefits of corticosteroid injection compared to anti-inflammatory medications, with 92% of individuals reporting absent pain or significantly improved pain at 4 weeks after corticosteroid injection compared to 57% of individuals undergoing a trial of naproxen and 50% of individuals with placebo. These same cohorts of patients demonstrated no difference in pain control and outcomes at 12 months [8]. More recent studies have suggested that corticosteroid injections offer only short-term relief and that these patients may have more pain and dysfunction at longer follow-up compared to other patients treated with conservative measures. Smidt and Bisset performed studies using corticosteroid injections, physical therapy, and the “wait and see” approach and found that steroid injections are most effective at 6 weeks. However at longer follow-up near one year, there were no significant differences between physical therapy and the “wait and see” approach whereas the injection cohort has significantly more pain and dysfunction comparatively [21, 22, 60]. Recently, authors have also noted potential long-term adverse effects of corticosteroid use for lateral epicondylitis including decreased tenocyte replication and collagen production [6]. Additionally, there have been reports of common extensor tendon rupture following corticosteroid injection [61]. Overall, steroid injections may provide a beneficial effect on pain in the short term (under 6 weeks), but there is no evidence that patient outcomes are improved with steroid injections beyond 6–8 weeks, and in many studies, those receiving injections have inferior long-term results compared to placebo.

### 6.2. Prolotherapy

Prolotherapy for lateral epicondylitis includes multiple injections of a small amount of irritant or sclerosing solution over the course of a two-week trial. Commonly used irritants include hypertonic dextrose, phenol-glycerine-glucase, or sodium morrhuate [62]. The proposed mechanism of prolotherapy injections is that the hypertonic dextrose causes cell rupture through osmosis while the monosodium morrhuate attracts inflammatory mediators and improves blood supply to the diseased tendon [63]. Scarpone and colleagues [64] performed a randomized controlled trial comparing prolotherapy consisting of hypertonic dextrose and sodium morrhuate versus placebo for lateral epicondylitis. A series of 3 separate injections were performed over 8 weeks and those patients in the prolotherapy group had significantly improved pain scores and isometric strength at 16 weeks compared to placebo. No long-term data suggests that prolotherapy allows for better pain relief and function compared to placebo and further long-term follow-up studies are needed for better recommendations.

### 6.3. Nitric Oxide

Nitric oxide (NO) is a soluble gas in the family of enzymes called nitric oxide synthases (NOSs). Nitric oxide exists as a free radical and can be toxic, but in smaller doses it acts as a messenger and is important in blood flow regulation. In normal uninjured, undamaged tissue, there is very little role for NOS; however there are much higher levels of NOS activity within the healing tendon in many animal models [65]. NOS is likely important during tendon healing related to its regulation of local blood flow and host defense. Some authors suggest that NO is also important for collagen synthesis, which is critical to healing within the tendinotic region in processes like lateral epicondylitis [66]. For this reason, NO has been applied for the treatment of tennis elbow and clinical trials have shown that when NO is delivered via a transdermal patch, there is reduction in pain, increase in range of motion, and increased strength. Paoloni and colleagues have performed numerous studies utilizing topical nitric oxide (glyceryl trinitrate) along with standard rehabilitation programs and they found that compared to a group that received a placebo patch, the patients in the glyceryl trinitrate group had significantly less elbow pain and tenderness for up to 12 weeks as well as increased wrist extensor peak forces at 6 months after treatment [67–69]. A follow-up randomized controlled trial confirmed that 8 weeks of glyceryl trinitrate patch application led to significantly less elbow pain with activity compared to a placebo patch application [69]. However, a more recent longer term follow-up demonstrated that the effects of topical glyceryl trinitrate appear to be more short lived, with very little effect demonstrated after the 6-month period and no real difference in clinical outcomes at 5 years compared to a group undergoing a standard tendon rehabilitation program without any other treatment [67].

## 7. Conclusions

Lateral epicondylitis is a common and disabling condition that leads to pain, time away from work, and difficultly with activities of daily living. Many different types of treatments have been investigated to help patients relieve pain and resume function more quickly while also helping to facilitate tendon healing. Recent studies within the field of biologics, including PRP, ABI, and stem cell therapies, have shown some promising results with minimal side effects. Future developments in the use of PRP will likely require a standardization protocol of PRP preparation prior to implementing large scale studies and recommendations for use. Early use of stem cell therapies for lateral epicondylitis has shown some promise, but most of the studies conducted have been in animal models. Prospective clinical studies are needed to determine the effectiveness while also examining the risks associated with stem cell use. Nonbiologic therapies have also proved to be effective in some clinical studies. While all of these treatment modalities represent potentially exciting management options for lateral epicondylitis, further long-term studies are needed to demonstrate the safety and efficacy of these therapies.

## Figures and Tables

**Table 1:** Summary of studies of biologics for lateral epicondylitis.

Author	Type of study	Level of evidence	Number of participants	Primary outcome measures	Results
Edwards and Calandruccio [26]	Case series	IV	28 patients were injected with 2 mL of autologous blood under ECRB; 19/28 patients received 1 injection; 7/28 patients received 2 injections; 2/28 patients received 3 injections	0–10 patient ratings of pain; patient rating according to Nirschl staging (0–7)	79% of all patients with complete resolution of pain at average of 9.5 months of follow-up; reduction in pain score from mean of 7.8 to 2.3 at last follow-up; reduction in Nirschl staging from mean of 6.5 to 2.0 at last follow-up

Mishra and Pavelko [40]	Cohort	II	15 treated with PRP; 5 treated with bupivacaine	VAS for pain	60% improvement in VAS with PRP versus 16% improvement in controls at eight weeks (*P* = 0.001); PRP group had 93% reduction in pain at final follow-up compared to baseline (*P* < 0.0001)

Connell et al. [51]	Case series	IV	12 treated with injection of collagen-producing tenocyte-like cells derived from autologous skin fibroblasts	PRTEE	Significant improvement in PRTEE scores at 6 weeks, 3 months, and 6 months compared to baseline; significant improvement in tendon structure noted on ultrasound

Peerbooms et al. [42]	RCT	I	51 treated with PRP; 49 treated with corticosteroid	Successful outcome: >25% reduction in VAS or DASH score without reintervention	73% successful outcome in PRP group versus 49% successful outcome in corticosteroid group based on VAS (*P* < 0.001) at 1 year; 73% successful outcome in PRP group versus 51% successful outcome in corticosteroid group based on DASH score (*P* = 0.005) at 1 year

Creaney et al. [27]	RCT	I	70 treated with PRP; 60 treated with ABI	Improvement in PRTEE score >25 points at final follow-up	No significant difference in success rates between PRP and ABI groups at 6 months (*P* = 0.59)

Gosens et al. [43]	RCT	I	51 treated with PRP; 49 treated with corticosteroid	Successful outcome: >25% reduction in VAS or DASH score without reintervention	77% successful outcome in PRP group versus 43% successful outcome in corticosteroid group based on VAS at 2 years (*P* < 0.0001); 73% successful outcome in PRP group versus 39% successful outcome in corticosteroid group based on DASH score at 2 years (*P* < 0.0001)

Wolf et al. [28]	RCT	II	9 treated with ABI and lidocaine; 9 treated with steroid and lidocaine; 10 treated with saline and lidocaine	DASH scores: PRFE pain and function scores; VAS score	No significance in DASH scores, PRFE pain scores, and VAS scores between the three groups at 2 months or 6 months of follow-up; PRFE function score significantly better for saline compared to PRP group at 6 months

Thanasas et al. [45]	RCT	I	14 treated with PRP; 14 treated with ABI	VAS for pain; Liverpool elbow score	PRP group had significantly greater improvement in VAS for pain at 6 weeks compared to ABI group (*P* < 0.05); no significant difference in VAS for pain at 3 months and 6 months; no significant differences in Liverpool elbow score at all time points

Krogh et al. [44]	RCT	I	20 treated with PRP; 20 treated with glucocorticoid; 20 treated with isotonic saline	Pain reduction at 3 months based on PRTEE pain score	No significant difference in pain reduction between PRP group, glucocorticoid group, and saline group at primary endpoint of 3 months (*P* > 0.05)

Wang et al. [18]	Case series	IV	16 treated with injection of autologous tenocytes derived from patellar tendon cells	VAS, Quick-DASH, grip strength, and grade of tendinopathy at common extensor origin on MRI	Significant improvement at 1, 3, 6, and 12 months in mean VAS, Quick-DASH scores, and grip strength; significant improvement in grade of tendinopathy on MRI at 12 months

Mishra et al. [41]	RCT	II	116 treated with PRP; 114 treated bupivacaine	Successful outcome: improvement of >25% in VAS with resisted wrist extension; patient-reported elbow tenderness	No significant difference in successful outcomes or percentage reporting elbow tenderness at 12 weeks; success rate of 83.9% in PRP group versus 68.3% in control group at 24 weeks (*P* = 0.037); significant elbow tenderness reported in 29.1% of PRP group versus 54.0% of control group at 24 weeks (*P* = 0.009)

Raeissadat et al. [46]	RCT	I	33 treated with PRP; 31 treated with ABI	VAS, pressure pain threshold, modified Mayo clinic performance index	Significant improvement in both groups in all pain variables compared to baseline at 4 weeks, 8 weeks, 6 months, and 12 months; no significant differences between ABI and PRP groups at any follow-up point

ABI = autologous blood injection; PRP = platelet rich plasma;

DASH = Disabilities of the Arm, Shoulder, and Hand;

PRFE = Patient-Rated Forearm Evaluation;

PRTEE = Patient-Rated Tennis Elbow Evaluation;

RCT = randomized controlled trial;

VAS = visual analog scale.

Note: trials of Peerbooms et al. [42] and Gosens et al. [43] include the same patient cohort with different time points of follow-up.
